# Therapeutic difficulties in a patient with Ehlers-Danlos syndrome and numerous symptomatic premature ventricular contractions—case report and literature review

**DOI:** 10.3389/fcvm.2023.1171541

**Published:** 2023-07-12

**Authors:** Grzegorz Sławiński, Elżbieta Wabich, Maja Hawryszko, Ludmiła Daniłowicz-Szymanowicz, Philippe Chevalier

**Affiliations:** ^1^ Department of Cardiology and Electrotherapy, Medical University of Gdańsk, Gdańsk, Poland; ^2^ Club 30, Polish Cardiac Society, Warsaw, Poland; ^3^ Service de Rythmologie, Centre de Référence National des Troubles du Rythme Cardiaque d’Origine Héréditaire de Lyon, Hôpital Cardiologique Louis Pradel, Hospices Civils de Lyon, Université de Lyon, Lyon, France

**Keywords:** Ehlers Danlos syndrome, arrhythmia, flecainide, ablation, pharmacotherapy

## Abstract

A 28-year-old female patient diagnosed with Ehlers-Danlos syndrome type III (hypermobile EDS, hEDS) was admitted to the cardiology clinic due to a 3-year history of symptomatic ventricular arrhythmia in the form of multiple premature ventricular contractions (PVCs). Attempts at antiarrhythmic treatment with beta-blockers, propafenone, and verapamil were unsuccessful. Due to the diagnosis of hEDS and the high risk of vascular complications related to the ablation procedure, invasive treatment was abandoned, and it was decided to implement flecainide. After the flecainide treatment initiation, a spectacular improvement in the number of ventricular arrhythmias was observed, along with the disappearance of the complaints previously reported by the patient. To the best of our knowledge, this is the first described case of spectacular flecainide antiarrhythmic effect in a patient with numerous PVCs also diagnosed with EDS. Flecainide treatment in the EDS group could be a successful alternative to ablation, which can lead to serious vascular and even life-threatening complications, especially after the failure of propafenone and beta-blockers treatment.

## Case report

**Figure 1:**
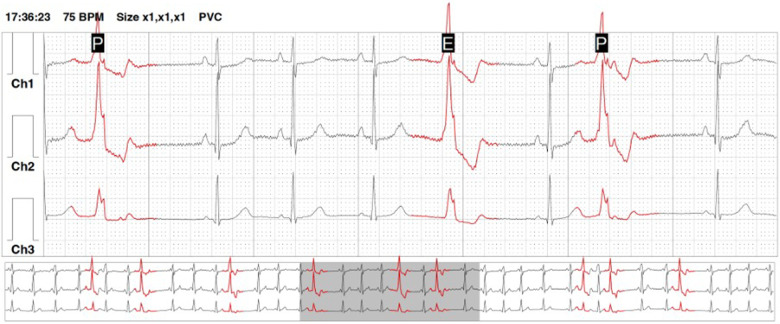
An example of a holter-ECG recording showing premature ventricular contractions arranged into ventricular bigeminy episodes.

**Figure 2:**
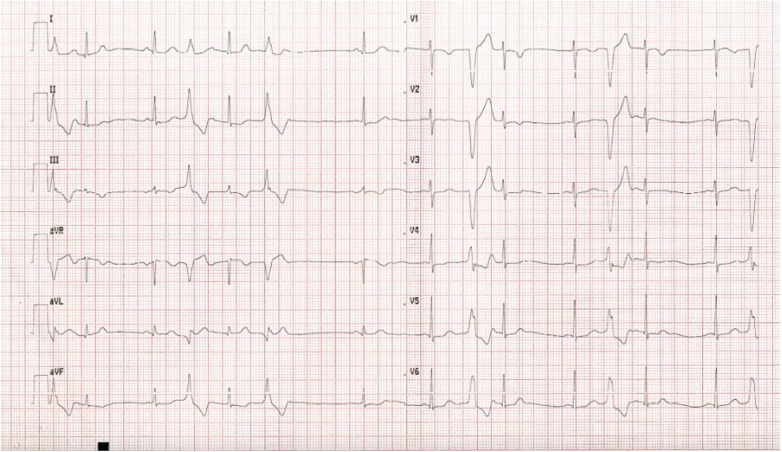
A 12-lead ECG showing ventricular premature contractions with right ventricular outflow tract morphology forming a ventricular bigeminy.

**Figure 3:**
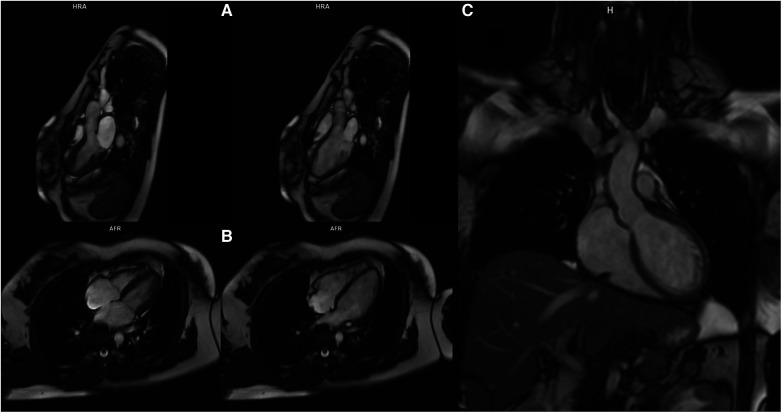
Magnetic resonance of the heart. Panel (**A**) End-systolic (left) and end-diastolic (right) still frame of the 3-ch cine steady state free precession series showing normal chamber size and no significant abnormalities of the valves. Panel (**B**) End-systolic (left) and end-diastolic (right) still frame of the 4-ch cine steady state free precession series normal chamber size and no significant abnormalities of the valves. Panel (**C**) Single shot steady state free precession coronal image showing both LVOT and ascending aorta.

A 28-year-old female patient diagnosed with Ehlers-Danlos syndrome type III (hypermobile EDS, hEDS) was admitted to the cardiology clinic because of a 3-year history of ventricular arrhythmia in the form of multiple premature ventricular contractions (PVCs). Previous transthoracic echocardiography revealed no abnormalities, except for the floppy anterior mitral valve leaflet accompanied by mild mitral regurgitation (Supplementary Videos S1–S3). The patient experienced ventricular arrhythmia as palpitations and stabbing chest pain. She also reported an exercise tolerance deterioration, periodic presyncope, and 3 syncope in the past. Due to the symptomatic arrhythmia, she has been treated initially with bisoprolol, and further with metoprolol succinate 25 mg daily and propafenone 150 mg twice daily. Regardless the treatment, Holter recordings repeatedly revealed numerous PVCs (from 6 to 20 thousand per 24 hours registration), often arranged into ventricular bigeminy and trigeminy episodes (Figure 1). PVCs were then evenly distributed during registration, with one morphology. The ECG performed during the consultation showed a sinus rhythm of 75 bpm and PVCs with a morphology pointing to the right ventricle outflow tract (RVOT) origin (Figure 2), which could indicate a potential possibility of radiofrequency ablation (RF) using with a positive effect. Unfortunately, this form of therapy, despite the fact that it's a typical treatment for such ventricular arrhythmia in the general population, in the case of our EDS patient was not appropriate to implement. Due to that fact, it was decided to escalate the current antiarrhythmic pharmacotherapy to 150 mg 3 times a day propafenone and 2  ×  25 mg metoprolol succinate. Additionally, cardiac magnetic resonance imaging was performed, excluding mitral valve prolapse and mitral annulus disjunction (Figure 3). In the control Holter-ECG which was performed after three months of the abovementioned therapy, no reduction in the number of PVCs was found and the patient was still very symptomatic. Further, it was decided to discontinue the current antiarrhythmic pharmacotherapy and start with verapamil at a dose of 40 mg three times a day. After another three months, there was no reduction in the number of PVCs in the next control Holter-ECG recording. We decided to start with flecainide at a dose of 2  ×  100 mg and metoprolol succinate at a dose of 25 mg once a day. After three months of the abovementioned therapy, a spectacular reduction in ventricular arrhythmia number was reached with only 3 PVCs in the periodic Holter-ECG registration. The symptoms previously reported by the patient were also resolved.

## Discussion

### EDS—pathophysiology and clinical manifestations

Ehlers–Danlos syndrome (EDS) is a group of genetically determined diseases caused by a collagen defect and metabolic disorders that lead to changes in connective tissue. The clinical spectrum ranges from mild skin and joint symptoms to severe physical disabilities, life-threatening complications, and sudden death (1). The diagnosis of many mutations resulted in the division of the disease into 14 syndromes (for instance, classic, vascular, with excessive joint mobility, cardiovascular, with dysplasia of the spine and hands, with muscle contractures, kyphoscoliotic, with corneal fragility syndrome, and others) differing in the clinical picture, frequency, and mode of inheritance (2). Known patterns of inheritance are autosomal dominant or recessive; some cases are also the result of spontaneous mutations (2).

Many genes are involved in EDS, but there is no current lack of reliable genotype–phenotype correlations. The most common are mutations in the collagen genes, leading to changes in the structure or a reduction in its amount (1).

The incidence of EDS is approximately 1 in 5,000 births, with the most common type of disease, hypermobile EDS (hEDS), accounting for 80%–90% of all diagnoses (3). Classic EDS (cEDS) accounts for about 1 in 20,000 births, while vascular EDS (vEDS), which is the most dangerous, is much less common, ranging from 1 in 50,000 to 1 in 200,000 births (4, 5). hEDS is inherited in an autosomal dominant fashion (6). The exact cause of the disease is unknown, but some cases are thought to be caused by a mutation in the tenascin-X (TNXB) gene (7). This type is characterized by complications with the digestive system, such as difficulty swallowing, and a slowed stomach and colon, resulting in absorption problems and food intolerance. Autonomic disorders, such as body temperature regulation, bowel and bladder function, and orthostatic intolerances affecting heart rate and blood pressure, have also been observed (2).

cEDS is inherited autosomal dominant (6). Its exact cause is the mutation of genes COL5A1 and COL5A2, which are responsible for maintaining the structure of V-type collagen (8). The disease is characterized by generalized joint hypermobility. Related complications may include numerous dislocations and sprains, curvature of the spine, flat feet, and premature degenerative joint diseases. The skin is thin and excessively stretchy, often with visible varicose veins. The patient may also develop “spontaneous” pneumothorax, changes in the heart valves, and neurological disorders (9). In pregnant women, there is a risk of uterine rupture and loss, which may lead to premature birth (8). The most severe vascular form of Ehlers–Danlos syndrome is inherited in an autosomal dominant manner. The exact cause is a mutation in the COL3A1 gene that causes changes in type III collagen (6). It is associated with a constant threat to life due to possible cardiovascular complications, such as ruptures of the walls of large vessels, including the aorta (10). Aneurysms are also common, especially in the abdominal aorta and cerebral arteries. There is also a risk of rupture of the gastrointestinal wall. In this form, any surgical procedure and more invasive diagnostic examination are at risk. Excessive joint mobility in this form is usually limited to small joints, most often fingers. The skin is thin and transparent, with a visible vascular system and it is easy to develop subcutaneous hemorrhages (9). Patients have a distinctive appearance: thin face, bulging eyes, pointed nose, increased amount of skin on the eyelids, and pale and prematurely aged appearance (1).

EDS is a relatively rare disease; the patient group exhibits phenotypic variation and overlapping symptoms, and the diagnostic criteria are imprecise. Therefore, making an accurate diagnosis is difficult (11).

### EDS—diagnostic criteria

Based on the 2017 international classification of EDS, there are 13 subtypes of EDS (12). In addition to molecular diagnostics, the authors of the above classification list individual clinical variables present in given subtypes of EDS. For the most common subtype—also present in the presented case report, i.e., hypermobile—the diagnosis remains clinical, as there is yet no reliable or appreciable genetic etiology to test for in most patients. The clinical diagnosis of hEDS requires the simultaneous presence of all three major criteria:

– Generalized joint hypermobility
– Two out of three: (1) systemic manifestations of a more generalized connective tissue disorder; (2) positive family history, with one or more first-degree relatives independently meeting the current diagnostic criteria for hEDS; and (3) musculoskeletal complications
– No features other forms of EDS or other alternative diagnoses met

**Figure 4:**
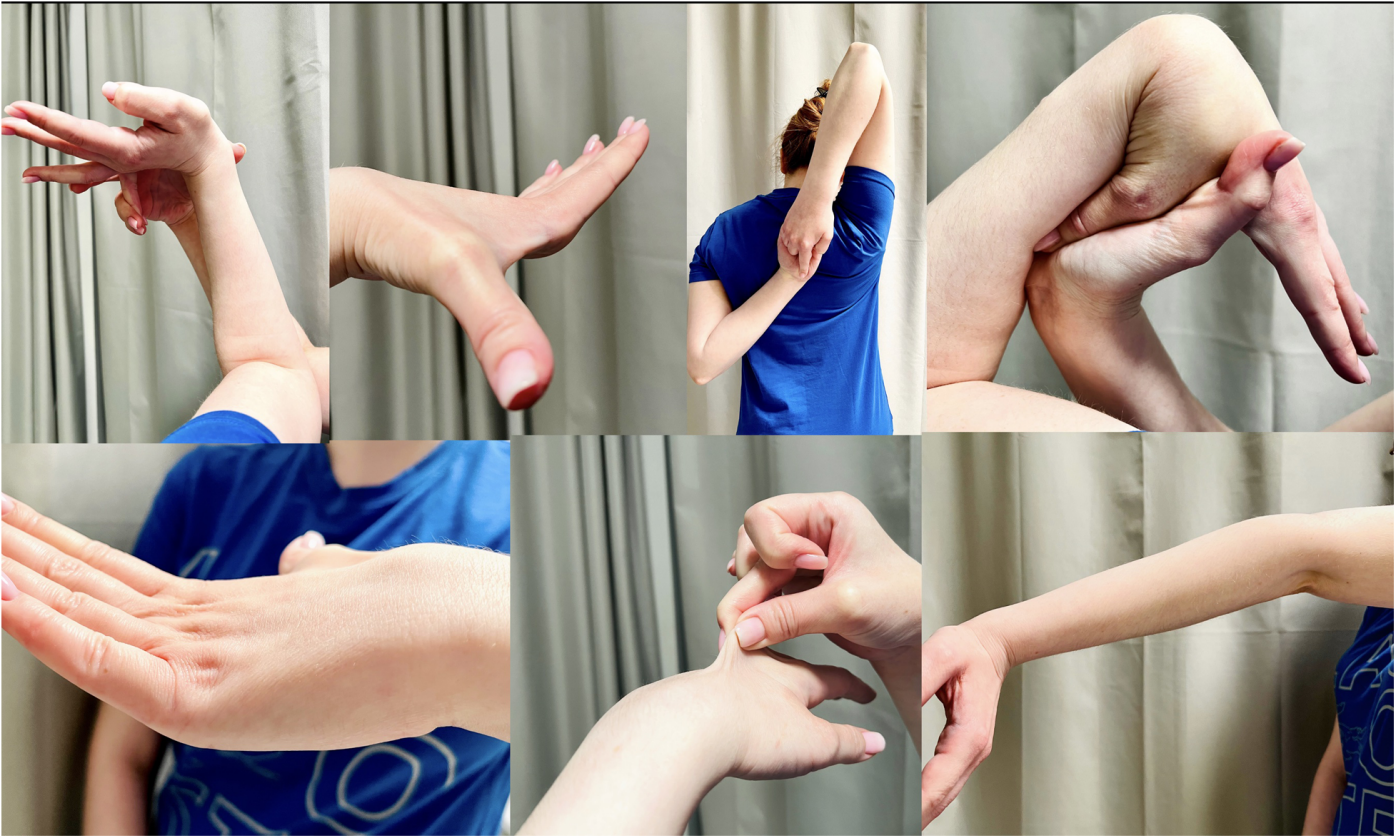
Clinical presentation of generalized joint hypermobility in reported patient.

In the case of our patient, hEDS was diagnosed based on generalized joint hypermobility, positive family history (mother of a patient diagnosed with EDS), musculoskeletal complications, features of chondropathy in the patellofemoral joint, and chronic and widespread pain for ≥3 months (Figure 4). The genetic studies performed so far have not been conclusive and the diagnosis of hEDS in the presented patient is based on clinical. In this case, the pain concerned mainly the right and left patellofemoral joints, sternal insertions of the ribs, both feet—in the metatarsal and calcaneal tubercles, and in the thoracic spine at the level of Th1-Th3. It should be emphasized that the assessment of the mobility of individual joints should be carried out strictly in accordance with the recommendations of the “2017 international classification of the Ehlers-Danlos syndromes”—while in Figure 4, the photos presented show generalized excessive skin extensibility and abnormal mobility in the patient's joints (12). The patient was repeatedly consulted with endocrinology, orthopedics, genetics, internal medicine, rheumatology, and other causes of reported ailments were excluded.

### Arrhythmias—prevalence and treatment

Although the presence of dysautonomia or valvular disease in EDS is well proven, there is no clear evidence (basically based on a few case reports) in the literature between the occurrence of arrhythmias and its prevalence in EDS (13–15). As early as 1977, one of the first case reports of a patient with EDS and accompanying cardiovascular disorders, including frequent premature ventricular contractions, was published (16). Other authors also presented case reports of patients with EDS and ventricular arrhythmias, including non-sustained ventricular tachycardia (17).

In a Camerota et al. study—24 hour Holter-ECG recordings were performed, involving 28 patients with joint hypermobility syndrome (JHS) and hEDS. In the analyzed group of patients, runs were found among arrhythmias: paroxysmal supraventricular tachycardia (4 patients), episodes of the sudden increase in heart rate (3 patients), and sinus arrhythmia (3 patients). However, there were no ventricular arrhythmias (18). In addition to the abovementioned, based on the data from the literature, atrio-ventricular nodal re-entry tachycardia (AVNRT), atrioventricular re-entry tachycardia (AVRT), and atrial fibrillation (AF) have also been found in patients with EDS (14, 19, 20).

Unfortunately, detailed data on the frequency of arrhythmias in EDS patients are not unknown, the reports from various authors are often divergent. For instance, in a study by Celletti et al. the aim was to assess cardiovascular autonomic involvement in JHS/EDS-HT by functional tests, which included thirty-five patients—the authors reported that none of the patients had cardiac arrhythmias (21).

Arrhythmias in patients with EDS may result, among others, from the presence of arrhythmic mitral valve prolapse (MVP), which is associated with a higher incidence of ventricular arrhythmias (14). In the case of our patient, no MVP was found, therefore it should be concluded that the arrhythmia was a clinical problem independent of EDS, which required consideration of an alternative treatment method to RF ablation.

Also, literature data concerning the usage of antiarrhythmic drugs in EDS patients are limited and based mainly on case reports or case series. In the abovementioned studies, the drug of the first choice used in antiarrhythmic therapy in patients with EDS is beta-blocker which was in line with our described treatment. Case reports of metoprolol treatment of paroxysmal atrial fibrillation are also available (20). Chronic amiodarone therapy as an alternative to invasive arrhythmia treatment with RF ablation—due to the high risk of further perforations of cardiac structures—could be also used, as in the general population, with good therapeutic effect (13). To the best of our knowledge, the data which considers the usefulness of class IC of antiarrhythmic drugs in EDS is unavailable. In the abovementioned case report, the first applied drug from this class, regardless of lower effectiveness, was propafenone, only due to the unavailability of flecainide in Poland. Flecainide, as a representative of class IC of antiarrhythmic drugs, is characterized by the highest efficiency, especially in sinus rhythm restoration. Flecainide's satisfactory effect on reducing ventricular arrhythmias is also well known. Despite the few side effects (especially QRS prolongation and negative inotropic effect), flecainide is described as the safest antiarrhythmic drug (along with propafenone) in the Cochrane database (22). Although propafenone and flecainide belong to class Ic antiarrhythmic drugs according to the Vaughan-Williams Classification and have a very similar mechanism of action, their metabolism differs, which is particularly pronounced depending on whether the patient is an extensive metabolizer or a poor metabolizer in the cytochrome P450 (CYP) 2D6. There is a clear gene-concentration relationship for propafenone due to polymorphic CYP2D6-mediated 5-hydroxylation. In extensive metabolizers, the pharmacokinetics of propafenone are nonlinear because of saturation of metabolism. Since flecainide is mainly eliminated through renal excretion, and both R- and S-flecainide possess equivalent potency for sodium channel inhibition, the CYP2D6 phenotype has a minor impact on the response to flecainide. Additionally, CYP2D6 polymorphism influences flecainide disposition and ECG effects only after a single dose but not at steady state (23).

The problem of arrhythmias was also observed among patients with another genetic connective tissue disorder–Loeys-Dietz Syndrome. In the case report described by Jaramillo on premature ventricular contractions, similar to ours, pretreatment with beta and calcium channel blockers was also unsuccessful and RF ablation was eventually performed (24). A rare coincidence of EDS and Brugada Syndrome was described by D'Souza et al. when a patient was treated with chronic quinidine and underwent an implantable cardioverter defibrillator implantation (25).

Due to the possibility of potential complications, RF ablation is usually not the first-choice method in the treatment of patients with EDS and accompanying cardiac arrhythmias. Patients with type IV EDS are at the highest risk of blood vessels perforation during invasive procedures. Confirmation of the increased risk of RF ablation in this group of patients is the Official Report of the Spanish Society of Cardiology Working Group on Electrophysiology and Arrhythmias published by the Spanish Catheter Ablation Registry. In this registry, only 4 deaths were reported as complications of ablations (out of 7,840 procedures performed), one of which occurred after an accessory pathway ablation, due to aortic rupture at 48-hour post procedure in a patient with EDS (19). Nevertheless, data on effective RF ablations in this group of patients are also available in the literature. Szili-Torok et al. described a case report of RF ablation for typical atrio-ventricular nodal re-entry tachycardia (AVNRT) in a patient with type IV EDS. RF ablation of the AVNRT was undertaken using the magnetic navigation system, with a non-traumatic single floppy catheter and advanced navigation capability. There were no complications related to the procedure and at 6-month follow-up, the patient was completely free from arrhythmias (14). Case reports describing RF ablation of ventricular tachycardia (VT) in EDS patients are also available. Calvert et al. in their study suggest the advantage of transvenous access with subsequent transseptal puncture under the control of transesophageal echocardiography (TEE), rather than retrograde aortic approach due to arterial fragility and risk of aortic dissection. Intravenous access was obtained under ultrasound control, for cardiac pacemapping, the authors used CARTO 3D navigation (Biosense Webster Inc., Irvine, CA) and a multipolar PentaRay catheter (Biosense Webster Inc.). Ablation was undertaken at 40 W for approximately 45 s per lesion to homogenize the entire channel of late ripple activation. No complications were observed after the procedure, and the implantable cardioverter-defibrillator (ICD) control showed no recurrence of VT (15).

Of course, in order to assess the safety and effectiveness of a given drug, studies should be conducted on a cohort of patients with EDS and concomitant ventricular arrhythmias. The presented case report is only the authors' experience in the treatment of one patient with EDS, which in no way defines the effectiveness of flecainide treatment among patients with EDS.

## Conclusions

To the best of our knowledge, this is the first described case of spectacular flecainide antiarrhythmic effect in a patient with numerous PVCs also diagnosed with EDS. Flecainide in the EDS patients could be a successful, alternative to RF ablation, treatment, which can lead to serious vascular and even life-threatening complications in such patients. It is necessary to conduct studies on larger groups of patients to confirm the efficacy and safety of flecainide in patients with EDS.

## Data Availability

The raw data supporting the conclusions of this article will be made available by the authors, without undue reservation.
